# HER2-Low Versus HER2-Zero Breast Cancer in the Neoadjuvant Setting: Pathological Complete Response and Exploratory Survival Outcomes in a Single-Center Cohort

**DOI:** 10.3390/medicina62071261

**Published:** 2026-06-30

**Authors:** Ümitcan Ateş, Merve Keskinkılıç, Hatice Miraç Binnaz Demirkan

**Affiliations:** 1 Division of Allergy and Immunology, Department of Internal Medicine, Ege University Faculty of Medicine, 35100 Izmir, Türkiye; 2 Department of Medical Oncology, İzmir Demokrasi University Buca Seyfi Demirsoy Training and Research Hospital, 35390 Izmir, Türkiye; 3 Division of Medical Oncology, Department of Internal Medicine, Faculty of Medicine, Dokuz Eylül University, 35340 Izmir, Türkiye

**Keywords:** HER2-low, breast cancer, neoadjuvant chemotherapy, pathological complete response, trastuzumab deruxtecan, hormone receptor, overall survival, disease-free survival

## Abstract

*Background and Objectives*: The HER2-low designation has emerged as a clinically actionable category in breast cancer following the approval of trastuzumab deruxtecan for HER2-low metastatic disease. However, the clinical relevance of HER2-low expression in the neoadjuvant chemotherapy (NAC) setting remains uncertain. This study aimed to evaluate pathological complete response (pCR) as the primary endpoint and overall survival (OS) and disease-free survival (DFS) as exploratory secondary endpoints across HER2-low, HER2-zero, and HER2-positive subgroups in a NAC-treated cohort. *Materials and Methods*: This single-center retrospective cohort study included 118 patients with histopathologically confirmed invasive breast cancer who received NAC at our institution between January 2010 and March 2021. Patients were classified as HER2-zero (IHC 0, *n* = 66), HER2-low (IHC 1+ or IHC 2+/FISH-non-amplified, *n* = 17), or HER2-positive (IHC 3+ or IHC 2+/FISH-amplified, *n* = 35). pCR was defined as ypT0/Tis ypN0. Univariate analyses used χ^2^ or Fisher’s exact tests; multivariable logistic and Cox regression were performed for adjusted analyses, and Kaplan–Meier survival curves were compared by log-rank, Breslow, and Tarone–Ware tests. *Results*: The overall pCR rate was 24.6%, differing significantly across HER2 subgroups (HER2-positive 45.7%, HER2-low 29.4%, HER2-zero 12.1%; *p* = 0.001). After multivariable adjustment for age, ER, PR, and tumor grade, HER2-positive status retained an independent association with pCR (OR 4.37, 95% CI 1.46–13.10, *p* = 0.008), whereas HER2-low status did not (OR 2.65, 95% CI 0.60–11.75, *p* = 0.201). At a median follow-up of 48.8 months, neither OS (log-rank *p* = 0.567) nor DFS (log-rank *p* = 0.901) differed significantly across HER2 subgroups, and HER2 subgroup status was not independently associated with survival in exploratory Cox models. *Conclusions*: In this NAC-treated cohort, the unadjusted pCR advantage of HER2-low over HER2-zero tumors was not retained after adjustment for hormone receptor expression and tumor grade, and no HER2 subgroup-specific survival difference was demonstrated. Within the standard NAC framework, HER2-low disease did not show a pCR or survival pattern clearly distinct from HER2-zero disease after adjustment; the small HER2-low subgroup and wide confidence intervals preclude firm conclusions, and an exploratory hormone receptor-stratified analysis indicated that the apparent pooled HER2-low advantage was confined to the hormone receptor-negative subgroup. Future prospective studies powered for hormone receptor-stratified analyses are warranted.

## 1. Introduction

Breast cancer remains the most frequently diagnosed malignancy among women and a leading cause of cancer-related mortality worldwide. Over the past decade, advances in molecular tumor characterization have refined breast cancer treatment strategies and shifted clinical practice toward biomarker-driven decision-making. Within this context, HER2 expression has become a critical determinant of therapeutic eligibility, particularly with the emergence of HER2-low breast cancer as a clinically actionable category [1].

Traditionally, HER2 status has been dichotomized as HER2-positive or HER2-negative, with IHC 3+ or IHC 2+/FISH-amplified tumors classified as HER2-positive and all non-amplified tumors grouped as HER2-negative. However, tumors scored as IHC 1+ or IHC 2+/FISH-non-amplified, collectively referred to as HER2-low, have gained clinical relevance because they may differ from HER2-zero tumors in treatment eligibility and, in selected subgroups, clinical behavior [2].

The therapeutic relevance of the HER2-low designation was redefined by the DESTINY-Breast04 phase III trial. In this trial, trastuzumab deruxtecan (T-DXd) significantly improved progression-free and overall survival compared with physician’s choice chemotherapy in patients with HER2-low metastatic breast cancer. These findings established HER2-low status as an actionable therapeutic category rather than merely a pathological descriptor [3].

In the neoadjuvant setting, pathological complete response (pCR) after systemic therapy is widely recognized as a surrogate endpoint for long-term outcomes, particularly in HER2-positive and triple-negative breast cancer. The association between pCR and improved disease-free survival (DFS) and overall survival (OS) has been consistently demonstrated across multiple large-scale analyses [4]. Whether this predictive and prognostic framework applies equivalently to HER2-low tumors, however, remains an area of active investigation.

Accumulating data indicate that HER2-low tumors are more frequently hormone receptor-positive, have lower histological grade, and show lower Ki-67 indices than HER2-zero tumors, features that may be associated with reduced chemosensitivity. In a pooled individual-patient analysis of four prospective neoadjuvant clinical trials including 2310 patients, HER2-low tumors demonstrated a significantly lower pCR rate than HER2-zero tumors (29.2% vs. 39.0%, *p* = 0.0002), with the difference particularly pronounced in the hormone receptor-positive subgroup [5].

Several retrospective studies have examined pCR and survival in HER2-low breast cancer with varying conclusions. Won and colleagues, in a nationwide Korean cohort of 30,491 patients, found no significant difference in overall survival between HER2-low and HER2-zero groups, though HER2-low status was independently associated with better breast cancer-specific survival in the triple-negative subgroup [6]. Kang and colleagues, in a Korean single-center cohort of 1288 patients who underwent NAC, demonstrated substantial HER2 status transition between pre- and post-treatment specimens, with no significant association between HER2-low status and clinical outcomes except in the hormone receptor-negative subset [7]. In contrast, Zhao and colleagues, in a large Chinese cohort, reported that HER2-low status was independently associated with lower odds of pCR in the hormone receptor-positive subgroup, highlighting a potential subtype-specific modulating effect of HER2-low expression on NAC response [8].

Taken together, these findings suggest that the prognostic and predictive significance of HER2-low status in the neoadjuvant setting is heterogeneous and likely shaped by the broader hormonal and molecular context of the tumor. Real-world institutional data examining pCR, DFS, and OS across HER2 subgroups remain limited, particularly in NAC-treated cohorts. The present study therefore aimed to evaluate pCR as the primary endpoint and DFS and OS as exploratory secondary endpoints in a cohort of 118 breast cancer patients treated with neoadjuvant systemic therapy at our institution, comparing outcomes across HER2-low, HER2-zero, and HER2-positive subgroups.

## 2. Materials and Methods

### 2.1. Study Population and Design

This retrospective cohort study included 118 patients with histopathologically confirmed invasive breast cancer who received neoadjuvant systemic therapy and were treated at the Medical Oncology Department of Dokuz Eylül University Faculty of Medicine Hospital between January 2010 and March 2021.

#### 2.1.1. Inclusion Criteria

Histopathological confirmation of invasive breast cancer by core needle biopsy; early-stage or locally advanced breast cancer; receipt of neoadjuvant chemotherapy, with or without HER2-directed therapy where indicated; follow-up conducted at Dokuz Eylül University Faculty of Medicine Hospital; availability of complete medical records was required for HER2 classification, treatment response assessment, and survival analysis; age ≥ 18 years at diagnosis.

#### 2.1.2. Exclusion Criteria

Absence of surgical resection following neoadjuvant chemotherapy; prior history of any solid or hematological malignancy; presence of systemic distant metastasis at initial diagnosis.

Histopathological parameters were obtained from the Department of Medical Oncology archive records, and tumor staging was reassigned in accordance with the 2018 updated American Joint Committee on Cancer (AJCC) Breast Cancer Staging Guidelines, 8th edition [9].

### 2.2. Ethics Committee Approval

This study was conducted following approval by the Non-Interventional Research Ethics Committee of Dokuz Eylül University, granted on 14 September 2022, under file number 7453-GOA, in accordance with the Declaration of Helsinki. Because the cohort comprised patients treated between January 2010 and March 2021, ethics approval was obtained in 2022 specifically for the retrospective analysis of this already treated cohort; written informed consent was obtained from all patients who could be contacted, and the committee approved the use of de-identified records for the remaining patients.

### 2.3. Data Collection

Age at diagnosis was calculated using the date of birth and the date of pathological diagnosis. The date of diagnosis was defined as the date of pathological confirmation. The start date of neoadjuvant treatment was defined as the date of the first chemotherapy cycle, and the end date was defined as the date of the last NAC cycle. DFS was calculated from the date of surgery to the first documented recurrence or death from any cause. Post-operative pathological data were reviewed to determine the type of response to neoadjuvant therapy. Follow-up imaging results were reviewed to determine recurrence status and recurrence date. The date of last follow-up and date of death were used to calculate overall survival. Pre-treatment radiological findings and receptor status, including estrogen receptor (ER), progesterone receptor (PR), and HER2, were recorded for each patient. ER and PR expression levels were categorized as 0–10%, 10–50%, and ≥50%. Post-NAC pathological variables, including residual Ki-67, residual ER and PR expression, post-NAC tumor grade, post-NAC HER2 status, and residual disease localization, were also recorded where available. For patients experiencing recurrence, recurrence pattern (locoregional vs. distant), site of metastasis, type of breast and lymph node metastasis, and time to first recurrence from the date of surgery were documented.

### 2.4. HER2 Classification

HER2 status was determined on pre-treatment core needle biopsy specimens using immunohistochemistry (IHC) and, where indicated, fluorescence in situ hybridization (FISH), in accordance with the 2018 American Society of Clinical Oncology/College of American Pathologists (ASCO/CAP) guidelines for HER2 testing in breast cancer [10]. Patients were classified into three groups: HER2-positive (IHC 3+ or IHC 2+ with FISH amplification, *n* = 35), HER2-low (IHC 1+ or IHC 2+ with FISH non-amplification, *n* = 17), and HER2-zero (IHC 0, *n* = 66) [1]. In a subset of patients with residual disease following NAC (*n* = 30), post-treatment HER2 status was also recorded to evaluate HER2 expression dynamics.

### 2.5. Definition of Outcomes

Pathological complete response (pCR) was defined as ypT0/Tis ypN0, indicating no residual invasive tumor in the breast or axillary lymph nodes [4]. Radiological clinical response to NAC was categorized as complete response, partial response, or stable disease based on pre-operative imaging assessments, including breast MRI, ultrasonography, or mammography as available. Disease-free survival (DFS) was defined as the time from the date of surgery to the first documented recurrence event (locoregional or distant) or death from any cause, whichever occurred first. OS was defined as the time from the date of diagnosis to death from any cause or last known follow-up.

### 2.6. Statistical Analysis

All statistical analyses were performed using IBM SPSS Statistics version 24.0 (IBM Corp., Armonk, NY, USA). Following descriptive statistics, the Shapiro–Wilk test was applied to assess the distribution of continuous variables. Baseline and clinicopathological characteristics were primarily compared across HER2 subgroups and, where relevant, according to pCR and survival status.

Continuous variables were compared across the three HER2 subgroups using one-way analysis of variance for normally distributed variables and the Kruskal–Wallis test for non-normally distributed variables. When pairwise comparisons between two subgroups were performed, Student’s *t*-test or Mann–Whitney U test was used as appropriate; these pairwise analyses were considered exploratory, and no formal adjustment for multiple pairwise testing was applied. Results were expressed as mean ± standard deviation, median, and minimum–maximum values. Categorical variables were compared using the chi-square test and Fisher’s exact test, with Monte Carlo simulation (10,000 sampled tables, 99% confidence intervals) applied when expected cell counts fell below 5. Results were reported as frequencies and percentages. Overall survival and disease-free survival were estimated using the Kaplan–Meier method and compared between groups using the log-rank (Mantel–Cox), Breslow (Generalized Wilcoxon), and Tarone–Ware tests. Multivariable Cox regression models were constructed using clinically relevant covariates, including HER2 subgroup, age, ER expression, PR expression, tumor grade, and pCR achievement. Because pCR lies on the causal pathway between HER2 subtype and long-term outcome, it was included as a covariate in the survival models to estimate the direct effect of HER2 status independent of its effect mediated through pCR; a sensitivity Cox model omitting pCR was additionally fitted and is reported in Supplementary Table S1. Predictors of pCR were first evaluated by univariate logistic regression, followed by multivariable logistic regression modeling. All results were reported with 95% confidence intervals. A two-sided *p*-value of less than 0.05 was considered statistically significant throughout all analyses.

## 3. Results

### 3.1. Patient Characteristics

A total of 118 patients with invasive breast cancer who received NAC, with or without HER2-directed therapy where indicated, were included in the analysis. The median follow-up duration for the entire cohort was 48.77 months (range 12.03–136.47 months). Among patients who experienced recurrence (*n* = 26), the median time to recurrence from surgery was 15.18 months (range 2.80–106.17 months). A total of 26 patients experienced documented recurrence, and 29 DFS events occurred when deaths without documented recurrence (*n* = 3) were included. The median age at diagnosis was 50.98 years (range 27.03–82.55). According to HER2 classification, 66 patients (55.9%) were HER2-zero (IHC 0), 17 patients (14.4%) were HER2-low (IHC 1+ or IHC 2+/FISH-negative), and 35 patients (29.7%) were HER2-positive (IHC 3+ or IHC 2+/FISH-positive). Pathological axillary lymph node involvement at the time of surgery was present in 92% of patients. Vascular invasion was detected in 14% of patients, and surgical margin positivity was observed in 9%. Adjuvant endocrine therapy was administered to 72% of patients, and additional post-neoadjuvant systemic chemotherapy was administered in 5%. The mean number of excised lymph nodes was 10.98 (range 0–48), and the mean number of metastatic lymph nodes was 2.15 (range 0–17). The high pathological axillary nodal-positivity rate (92.4%) reflects the era-specific practice during the study period of reserving neoadjuvant chemotherapy largely for clinically node-positive or locally advanced disease; consequently, the cohort is enriched for higher-risk, node-positive tumors, which is consistent with the low overall pCR rate and should be considered when generalizing the findings.

### 3.2. Baseline Patient and Tumor Characteristics

#### 3.2.1. Tumor Grade

Histological grade distribution did not differ significantly across HER2 subgroups (*p* = 0.084). Grade 2 tumors predominated in the HER2-low group (62.5%), while grade 3 tumors were most frequent in the HER2-positive group (57.1%). No grade 1 tumors were identified in either the HER2-low or HER2-positive groups. In the HER2-zero group, 7.7% were grade 1, 58.5% grade 2, and 33.8% grade 3.

#### 3.2.2. Estrogen Receptor Expression

A statistically significant difference in ER expression distribution was observed across HER2 subgroups (Pearson χ^2^ = 14.526, *p* = 0.006; Fisher’s exact *p* = 0.003). Among HER2-low patients, 64.7% had ER expression ≥ 50%, while no patient in this group showed expression in the 10–50% range. In the HER2-zero group, 69.7% had ER ≥ 50% expression. The HER2-positive group had the lowest proportion of ER ≥ 50% expression (34.3%) and the highest proportion of ER 0–10% expression (45.7%) (Table 1).

#### 3.2.3. Progesterone Receptor Expression

PR expression also differed significantly across HER2 subgroups (Pearson χ^2^ = 13.926, *p* = 0.008; Fisher’s exact *p* = 0.002). The HER2-positive group had markedly higher PR negativity (0–10% category: 88.6%), with no patients showing PR expression in the 10–50% range. PR ≥ 50% expression was most common in the HER2-zero group (28.8%), followed by HER2-low (23.5%) and HER2-positive (11.4%) groups (Table 1).

### 3.3. Response to Neoadjuvant Chemotherapy

#### 3.3.1. Radiological Clinical Response

Radiological response differed across HER2 subgroups (Fisher’s exact *p* = 0.040), although this finding should be interpreted cautiously given the small subgroup sizes. Complete radiological response was most frequent in the HER2-positive group (40.0%), followed by HER2-low (35.3%) and HER2-zero (22.7%) groups. Stable disease was absent in the HER2-positive group, present in 5.9% of HER2-low patients, and most common in HER2-zero patients (16.7%). Partial response rates were comparable across the three groups (HER2-zero 60.6%, HER2-low 58.8%, HER2-positive 60.0%). The statistically significant difference in radiological response across subgroups did not translate into a fully concordant pathological (pCR) pattern. This is expected because radiological categories capture macroscopic tumor reduction and cannot resolve microscopic residual invasive disease; moreover, imaging was performed with heterogeneous modalities (MRI, ultrasonography, or mammography as available) without standardized central radiological review, which further limits the radiological–pathological correlation.

#### 3.3.2. Pathological Complete Response—Univariate Analysis

pCR rates differed significantly across HER2 subgroups on univariate analysis (Pearson χ^2^ = 14.175, *p* = 0.001; Fisher’s exact *p* = 0.001). The overall pCR rate in the cohort was 24.6% (29/118). The HER2-positive group achieved the highest pCR rate at 45.7% (16/35), followed by the HER2-low group at 29.4% (5/17), and the HER2-zero group at 12.1% (8/66). Residual disease was most prevalent in the HER2-zero group (87.9%), followed by the HER2-low group (70.6%) and the HER2-positive group (54.3%) (Table 2).

#### 3.3.3. Pathological Complete Response—Multivariable Logistic Regression

To evaluate whether HER2 subgroup status was associated with pCR after adjustment for clinically relevant covariates, a multivariable logistic regression analysis was performed including HER2 subgroup (reference: HER2-zero), age, ER expression category, PR expression category, and tumor grade as covariates. The analysis was conducted in 116 patients with complete covariate data (two patients excluded due to missing tumor grade), among whom 27 (23.3%) achieved pCR.

HER2-positive status was associated with higher odds of pCR after multivariable adjustment (OR 4.37, 95% CI 1.46–13.10, *p* = 0.008). Tumor grade showed a non-significant trend toward association with pCR (OR 2.63, 95% CI 0.97–7.14, *p* = 0.059). HER2-low status was not significantly associated with pCR after adjustment for ER, PR, and grade (OR 2.65, 95% CI 0.60–11.75, *p* = 0.201), suggesting that the numerically higher unadjusted pCR rate observed in HER2-low compared with HER2-zero tumors may be partly explained by differences in hormone receptor expression and tumor grade across HER2-defined subgroups rather than by HER2-low status itself. Age (OR 1.01, *p* = 0.750), ER expression (OR 0.65, *p* = 0.167), and PR expression (OR 0.62, *p* = 0.283) were not independently associated with pCR in the multivariable model (Table 3).

#### 3.3.4. Hormone Receptor-Stratified Pathological Complete Response (Exploratory)

To assess whether the pooled HER2-low versus HER2-zero comparison was confounded by hormone receptor status, pCR was examined within hormone receptor strata (hormone receptor-positive defined as ER or PR > 10%). The numerically higher unadjusted pCR rate of HER2-low tumors in the overall cohort was confined to the hormone receptor-negative subgroup. Among hormone receptor-positive patients, no HER2-low tumor achieved pCR (0/11, 0.0%), a rate numerically lower than that of HER2-zero tumors (4/53, 7.5%), whereas HER2-positive tumors achieved a markedly higher rate (9/19, 47.4%). Among hormone receptor-negative patients, pCR rates were 5/6 (83.3%) for HER2-low, 4/13 (30.8%) for HER2-zero, and 7/16 (43.8%) for HER2-positive tumors. Given the very small subgroup sizes, these results are strictly descriptive and hypothesis-generating (Table 4).

### 3.4. Survival Outcomes

The median follow-up duration was 48.77 months (range 12.03–136.47). Kaplan–Meier analyses of OS and DFS were performed as secondary exploratory analyses. Given the small number of events in each subgroup—particularly the HER2-low group (3 OS events, 4 DFS events)—these analyses have limited statistical power and the results should be interpreted with corresponding caution. All survival comparisons are presented without claims of independent prognostic attribution; multivariable Cox regression was performed to evaluate the adjusted effect of HER2 subgroup status on survival.

#### 3.4.1. Overall Survival—Kaplan–Meier Analysis

A total of 17 deaths (14.4%) occurred during follow-up; 101 patients (85.6%) were censored. The mean OS for the overall cohort was 111.5 months (95% CI: 101.1–121.8) and the median OS was 133.2 months (95% CI: 100.5–165.9).

**Figure 1:**
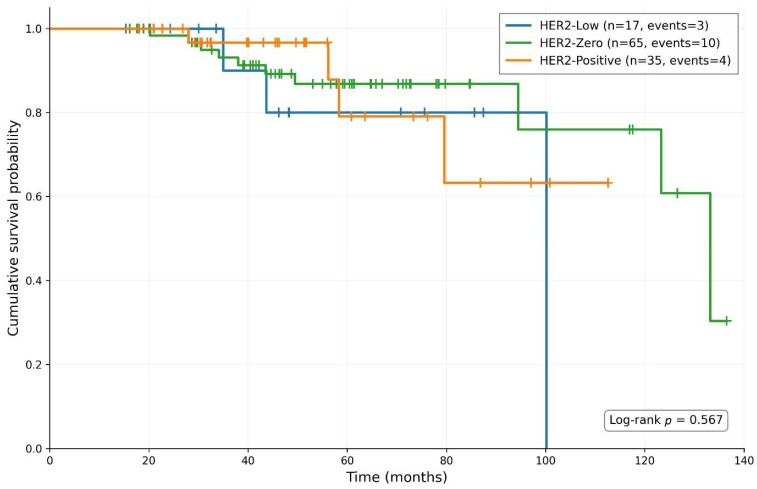
Kaplan–Meier overall survival curves by HER2 status. Log-rank *p* = 0.567 (Breslow *p* = 0.815; Tarone–Ware *p* = 0.775). Mean OS: HER2-low 88.0 months, HER2-zero 115.7 months, HER2-positive 94.8 months. Tick marks (+) indicate censored observations. Presented as exploratory secondary analysis; see Table 6 for multivariable Cox regression results.

When OS was compared across HER2 subgroups, the mean OS was 88.0 months (95% CI: 69.5–106.5) in the HER2-low group, 115.7 months (95% CI: 103.9–127.5) in the HER2-zero group, and 94.8 months (95% CI: 80.0–109.6) in the HER2-positive group. Median OS was 100.2 months in the HER2-low group and 133.2 months in the HER2-zero group; the median was not reached in the HER2-positive group due to the low number of events. Log-rank analysis revealed no statistically significant difference in OS between the three HER2 subgroups (log-rank χ^2^ = 1.133, *p* = 0.567; Breslow *p* = 0.815; Tarone–Ware *p* = 0.775) (Figure 1, Table 5).

**Table 6:** Multivariable Cox proportional hazards regression—overall survival and disease-free survival.

**Variable—Overall Survival (*n* = 115, Events = 17)**	**HR**	**95% CI**	***p***
HER2-Low vs. HER2-Zero	1.00	0.25–3.97	0.997
HER2-Positive vs. HER2-Zero	0.78	0.24–2.59	0.689
Age (years)	0.99	0.96–1.03	0.771
ER expression (ordinal)	0.51	0.22–1.19	0.120
PR expression (ordinal)	1.62	0.71–3.70	0.256
Tumor grade	0.86	0.32–2.31	0.768
pCR achievement	0.20	0.02–1.64	0.134
**Variable—Disease-Free Survival (*n* = 114, events = 29)**	**HR**	**95% CI**	***p***
HER2-Low vs. HER2-Zero	0.82	0.26–2.56	0.731
HER2-Positive vs. HER2-Zero	0.69	0.23–2.02	0.493
Age (years)	1.00	0.97–1.04	0.802
ER expression (ordinal)	1.04	0.57–1.90	0.897
PR expression (ordinal)	0.86	0.48–1.55	0.625
Tumor grade	0.70	0.32–1.55	0.383
pCR achievement	0.50	0.13–1.92	0.310

Reference category: HER2-zero. Covariates: HER2 subgroup, age, ER expression (ordinal), PR expression (ordinal), tumor grade, pCR achievement. OS: *n* = 115, 17 events. DFS: *n* = 114, 29 events. HR, hazard ratio; CI, confidence interval. Wide confidence intervals reflect limited event numbers.

Mortality rates were 17.6% (3/17) in the HER2-low group, 15.2% (10/66) in the HER2-zero group, and 11.4% (4/35) in the HER2-positive group. Chi-square analysis confirmed no significant difference in mortality between groups (*p* = 0.808).

#### 3.4.2. Disease-Free Survival—Kaplan–Meier Analysis

**Figure 2:**
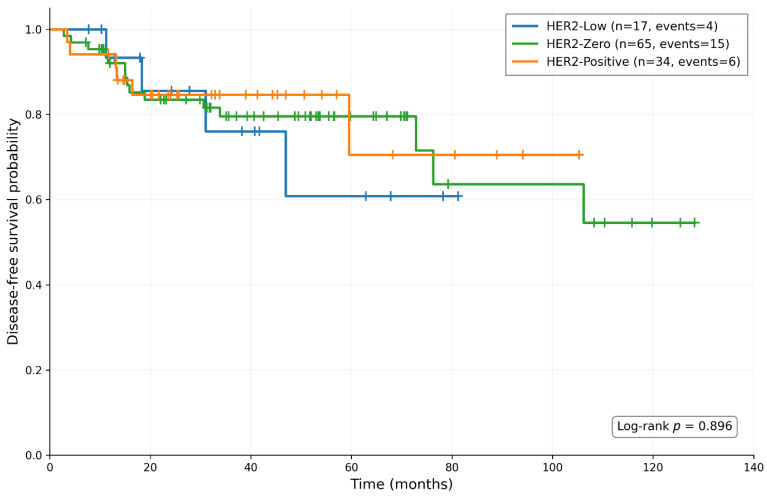
Kaplan–Meier disease-free survival curves by HER2 status. Log-rank *p* = 0.901 (Breslow *p* = 0.857; Tarone–Ware *p* = 0.945). Median DFS: HER2-low 35.0 months, HER2-zero 46.3 months, HER2-positive 46.3 months. DFS analysis includes all patients (events = 29). Presented as exploratory secondary analysis; see Table 6 for multivariable Cox regression results.

Among the 26 patients (22.0%) who experienced recurrence during follow-up, the median time to recurrence from surgery was 15.18 months (range 2.80–106.17 months; IQR 11.18–31.71 months). Kaplan–Meier analysis of DFS across HER2 subgroups demonstrated no statistically significant difference (log-rank χ^2^ = 0.208, *p* = 0.901; Breslow *p* = 0.857; Tarone–Ware *p* = 0.945). The mean DFS was 49.8 months (95% CI: 15.5–84.0) in the HER2-low group, 55.9 months (95% CI: 40.6–71.1) in the HER2-zero group, and 53.0 months (95% CI: 39.7–66.2) in the HER2-positive group. Median DFS was 35.0 months in the HER2-low group, 46.3 months in the HER2-zero group, and 46.3 months in the HER2-positive group (Figure 2, Table 5).

#### 3.4.3. Overall Survival—Multivariable Cox Regression

A multivariable Cox proportional hazards regression model for OS was constructed including HER2 subgroup (reference: HER2-zero), age, ER expression category, PR expression category, tumor grade, and pCR achievement as covariates. Three patients were excluded from the multivariable Cox model owing to incomplete covariate or follow-up data, resulting in a final analytical sample of 115 patients with 17 OS events.

In the exploratory multivariable Cox model, no variable was significantly associated with OS. HER2-low status was not significantly associated with OS compared with HER2-zero (HR 1.00, 95% CI 0.25–3.97, *p* = 0.997). HER2-positive status was similarly not significantly associated with OS (HR 0.78, 95% CI 0.24–2.59, *p* = 0.689). Among other covariates, ER expression showed a numerically protective but non-significant trend (HR 0.51, 95% CI 0.22–1.19, *p* = 0.120), and pCR achievement showed a numerically favorable but non-significant association (HR 0.20, 95% CI 0.02–1.64, *p* = 0.134). The absence of significant findings in this model reflects the limited number of OS events relative to the number of covariates included (Table 6). Inclusion of pCR as a covariate in the survival models attenuates any indirect HER2 effect mediated through pCR achievement; the HER2 hazard ratios estimated in these models should therefore be interpreted as the residual direct effect of HER2 status on survival after the mediating effect of pCR has been partialled out.

#### 3.4.4. Disease-Free Survival—Multivariable Cox Regression

A multivariable Cox regression model for DFS was similarly constructed using the same covariate set, including 114 patients with complete data and 29 DFS events.

In the exploratory multivariable Cox model, no variable was significantly associated with DFS. HER2-low status was not significantly associated with DFS compared with HER2-zero (HR 0.82, 95% CI 0.26–2.56, *p* = 0.731). HER2-positive status likewise showed no significant association with DFS (HR 0.69, 95% CI 0.23–2.02, *p* = 0.493). pCR achievement showed a numerically favorable but non-significant association with DFS (HR 0.50, 95% CI 0.13–1.92, *p* = 0.310). The wide confidence intervals throughout the DFS model reflect the relatively small number of events available for multivariable adjustment (Table 5).

### 3.5. Summary of Statistical Analyses

The main finding of this study was a significant difference in pCR rates across HER2 subgroups on univariate analysis (*p* = 0.001), with HER2-positive status remaining associated with higher odds of pCR in the multivariable logistic regression model (OR 4.37, *p* = 0.008). The numerically higher unadjusted pCR rate of the HER2-low group compared with HER2-zero was not confirmed as significant after multivariable adjustment (OR 2.65, *p* = 0.201); however, the wide confidence intervals leave open whether the loss of significance reflects genuine confounding by hormone receptor expression and grade, or the limited statistical power of the multivariable model. No HER2 subgroup effect on OS or DFS was observed on either univariate Kaplan–Meier or exploratory multivariable Cox regression analysis, consistent with the underpowered nature of the survival analyses given the small number of events (Table 7).

## 4. Discussion

This single-center retrospective study addressed whether HER2-low status identifies a distinct subgroup among patients with breast cancer treated with NAC when compared with HER2-zero and HER2-positive disease across pCR, OS, and DFS. Our findings suggest a consistent pattern, although interpretation is limited by the small HER2-low subgroup. Compared with HER2-zero tumors, HER2-low tumors did not differ significantly in pCR after adjustment for HR expression and tumor grade, and no clear OS or DFS difference was observed between these groups. However, because the survival analyses were limited by small event numbers, these null findings should be interpreted as hypothesis-generating rather than evidence of equivalence. Compared with HER2-positive tumors, HER2-low tumors achieved lower pCR rates, although the present cohort was underpowered to determine whether this difference translated into long-term survival differences.

After multivariable adjustment for hormone receptor expression and tumor grade, HER2-low disease did not behave distinctly from HER2-zero within the standard NAC framework, whereas HER2-positive disease retained an independent association with higher odds of pCR. The apparent unadjusted pCR advantage of HER2-low over HER2-zero tumors was no longer evident after adjustment for ER, PR, and tumor grade, representing the central analytical observation of our study. This finding suggests that the unadjusted HER2-low versus HER2-zero comparison should be interpreted with caution; however, the limited size of the HER2-low subgroup (*n* = 17) and the resulting wide confidence intervals leave open whether the loss of significance after adjustment reflects genuine confounding by hormone receptor expression and grade, or the limited statistical power of the multivariable model.

The direction of the unadjusted association in our cohort, in which HER2-low tumors achieved numerically higher pCR than HER2-zero tumors, differs from the largest pooled neoadjuvant dataset, in which HER2-low tumors had a significantly lower pCR rate than HER2-zero tumors in the HR-positive subgroup (17.5% vs. 23.6%) and no significant pCR difference in the HR-negative subgroup [5]; a similar HR-dependent suppression of pCR was reported in a Chinese neoadjuvant cohort [8]. The disappearance of the HER2-low signal after multivariable adjustment in our analysis is therefore most parsimoniously attributable to limited statistical power rather than to confounding by hormone receptor expression and grade alone. A Brazilian single-center cohort and a European translational dataset further confirmed that, when HR-positive or HR-negative subgroups are examined separately, HER2-low and HER2-zero patients respond comparably to standard NAC [2,11]. Mechanistically, this interpretation is plausible, as previous studies suggest that bidirectional crosstalk between ER signaling and the ERBB2 axis may contribute to low-level HER2 expression in luminal tumors without reaching the threshold of gene amplification. The clinical implication is straightforward: in the standard NAC setting, and in the absence of HER2-directed ADC therapy, clinicians should not anticipate a meaningfully different response between a HER2-low and a HER2-zero tumor of comparable HR phenotype and grade.

In contrast, HER2-positive status was associated with substantially higher odds of pCR in our cohort, consistent with the biology of HER2-overexpressing disease and with the documented surrogate value of pCR in HER2-positive subtypes [4]. However, this association must be interpreted in the context of HER2-directed neoadjuvant therapy administered where indicated, and the observed difference therefore reflects both biological subtype distinctions and treatment-related effects.

To assess whether the pooled HER2-low versus HER2-zero comparison was confounded by hormone receptor status, we performed an exploratory hormone receptor-stratified analysis of pCR (Table 4). The numerically higher unadjusted pCR rate of HER2-low tumors in the overall cohort was driven almost entirely by the hormone receptor-negative subgroup. Among hormone receptor-positive patients, HER2-low tumors achieved no pathological complete responses (0/11), a rate numerically lower than that of HER2-zero tumors (4/53, 7.5%), whereas among hormone receptor-negative patients HER2-low tumors showed a higher pCR rate (5/6) than HER2-zero tumors (4/13, 30.8%). Although based on very small subgroup numbers and therefore strictly hypothesis-generating, this pattern is directionally consistent with the published literature, in which HER2-low status has been associated with a significantly lower pCR rate specifically within the hormone receptor-positive subgroup in the largest pooled neoadjuvant dataset [5] and in subsequent population-based cohorts and meta-analyses [12,13,14,15]. The apparent reversal of this direction in our pooled analysis therefore most plausibly reflects confounding by hormone receptor distribution rather than a genuinely divergent biology, and underscores why hormone receptor-stratified, rather than pooled, comparisons are essential when evaluating the predictive relevance of HER2-low expression.

The absence of an OS difference between HER2-low and HER2-zero patients in our cohort is consistent with findings from several larger published series. A Korean nationwide registry of more than 30,000 patients reported no significant OS difference between HER2-low and HER2-zero patients in the unselected population, although it identified an independent breast cancer-specific survival advantage for HER2-low status specifically within the TNBC subgroup [6], and the European translational HR-positive cohort similarly found no OS difference [2]. A potentially divergent signal emerges only in HR-negative disease: in a Chinese TNBC neoadjuvant cohort, HER2-low patients had significantly longer OS than HER2-zero patients despite identical pCR rates [16], and the German pooled neoadjuvant analysis reported better 3-year OS for HER2-low within the HR-negative subgroup [5]. This subtype-specific signal is biologically intriguing because it suggests that low-level HER2 expression may carry prognostic information specifically when the tumor is not under hormonal influence—a context in which weak HER2 signaling may modulate immune microenvironment composition or differentiation programs in ways that are masked when ER signaling dominates tumor biology. Our cohort, which mixes HR-positive and HR-negative patients and contributes a very small number of OS events within the HER2-low subgroup, is not powered to detect a TNBC-specific survival difference of the magnitude reported in these larger cohorts, and our null OS finding for the HER2-low versus HER2-zero comparison should therefore be read as consistent with—rather than confirmatory of—the prevailing pattern in unselected populations.

The absence of an independent OS advantage for HER2-positive status in our Cox model warrants explicit comment because it diverges from the well-established OS benefit of trastuzumab-based therapy in adequately powered trials and pooled analyses [4]. This discrepancy does not imply biological equivalence between HER2-positive and HER2-low disease. Rather, it reflects the low event-to-covariate ratio in our survival models and the fact that our study period predates the routine clinical adoption of post-neoadjuvant T-DM1 for HER2-positive residual disease and of dual anti-HER2 neoadjuvant blockade as standard of care. The well-established overall survival benefit of anti-HER2 therapy in HER2-positive disease, supported by adequately powered randomized trials, is not contradicted by our null finding, which is more plausibly explained by the limited statistical power of our survival models.

The DFS findings recapitulate the OS pattern. We did not detect a significant DFS difference between HER2-low and HER2-zero patients, nor between HER2-low and HER2-positive patients, in either Kaplan–Meier or adjusted Cox analyses. The Brazilian cohort of HER2-zero NAC-treated patients similarly found no 5-year relapse-free survival difference between HER2-low and HER2-zero in either the luminal or TNBC subgroup [11], the Chinese neoadjuvant cohort reported no DFS difference overall or in either HR subgroup [8], and the European HR-positive cohort similarly found no event-free survival difference [2]. Once again, the only consistently divergent signal in the published literature is observed in HR-negative disease, where the German pooled neoadjuvant analysis reported significantly longer 3-year DFS for HER2-low than for HER2-zero patients overall and specifically within the HR-negative subgroup, with no DFS difference in the HR-positive subgroup [5]. The recurring pattern across both OS and DFS—a null signal in unselected and HR-positive populations but a possible survival advantage for HER2-low in HR-negative disease—is the single most clinically interesting feature of the HER2-low literature. Collectively, these findings suggest that HER2-low may not be a uniform biological entity and that HR-negative HER2-low disease may be the subgroup most likely to harbor a biologically relevant signal warranting dedicated prospective investigation. Our cohort cannot resolve this question because of its small HR-negative HER2-low component, but the limitation itself underscores why future studies must be powered for HR-stratified analyses rather than for overall comparisons.

The integrated picture across all three endpoints positions HER2-low disease, in the standard NAC framework, as clinically much closer to HER2-zero than to HER2-positive disease. This integrated picture contrasts with the metastatic setting, in which HER2-low status has been redefined as an actionable therapeutic target by the DESTINY-Breast04 phase III trial, where T-DXd significantly improved progression-free survival (median 9.9 vs. 5.1 months) and OS (median 23.4 vs. 16.8 months) compared with physician’s choice chemotherapy in previously treated HER2-low metastatic breast cancer [3]. This generates a clinically important distinction: a marker that does not appear to meaningfully influence response to standard NAC may nonetheless become therapeutically relevant in residual or recurrent disease, and patients with HER2-low residual disease after NAC may represent a clinically relevant population for future trials evaluating ADC-based post-neoadjuvant strategies.

Two pathology-related considerations follow directly from this paradox. First, HER2 status is not static across the course of NAC, with a substantial proportion of patients transitioning between HER2-low and HER2-zero categories in either direction during treatment, and ER-positive patients carrying significantly higher odds of acquiring HER2-low expression after NAC [7]. Second, recent assay-validation data suggest that some tumors historically scored as IHC 0 may be reclassified as HER2-low on central re-review with contemporary validated assays, indicating that the apparent HER2-zero population in historical cohorts may include patients with low-level HER2 expression [17]. The HER2 status determinations in our cohort were performed using historical assays in accordance with the 2018 ASCO/CAP focused update [10], but these assays were not specifically optimized for contemporary HER2-low assessment in the context of T-DXd eligibility, and a proportion of patients classified as HER2-zero in our cohort might be reclassified as HER2-low if archival material were re-evaluated with contemporary validated assays.

### Limitations

This study has several limitations. First, the HER2-low subgroup comprised only 17 patients, resulting in a critically small number of OS events (*n* = 3) and DFS events (*n* = 4), which preclude meaningful statistical inference about survival outcomes in this subgroup and substantially reduce the power of all multivariable analyses. Second, while multivariable logistic regression was performed for pCR and Cox regression for OS and DFS, the event-to-covariate ratios in the survival models are suboptimal (17 OS events with 7 covariates; 29 DFS events with 7 covariates), rendering the Cox models exploratory rather than confirmatory. Third, although a descriptive hormone receptor-stratified analysis of pCR is now provided (Table 4), the HER2-low subgroups within each hormone receptor stratum were too small (hormone receptor-positive, *n* = 11; hormone receptor-negative, *n* = 6) to support fully adjusted subgroup logistic models, so the independent effect of HER2-low status within hormonally defined contexts could not be formally estimated and the stratified estimates remain strictly descriptive [5]. Fourth, the retrospective design and the use of a single center’s data introduce selection and ascertainment bias. In addition, exclusion of patients who did not undergo surgical resection after NAC may have introduced selection bias, because patients with progression during NAC or poor treatment response may have been systematically excluded from the analysis. Fifth, the study period (2010–2021) predates the routine clinical adoption of T-DXd, post-neoadjuvant T-DM1 for HER2-positive residual disease, and dual anti-HER2 neoadjuvant blockade as standard of care, limiting the generalizability of survival findings to current practice. Sixth, three patients with incomplete covariate or follow-up records were excluded from the multivariable survival analysis. Finally, the median follow-up of 48.77 months may be insufficient to detect late recurrences in the hormone receptor-positive population, where recurrence risk extends characteristically beyond five years [4]. Furthermore, ER and PR expression were categorized as 0–10%, 10–50%, and ≥50%, which combines ER-negative and ER-low tumors within the lowest category and may reduce the granularity of adjustment for hormone receptor status. A further important limitation concerns potential HER2 misclassification: although HER2 status was determined in accordance with the 2018 ASCO/CAP focused update, the assays applied over the 2010–2021 period were not optimized for contemporary HER2-low assessment, and interobserver variability at the IHC 0 versus 1+ threshold, combined with the absence of central pathological re-review of archival material, may have produced classification bias directly affecting the central HER2-zero versus HER2-low comparison [12,17]. In addition, the type of surgical treatment (breast-conserving surgery versus mastectomy) was not modeled as a covariate, despite reported effects of surgical approach on long-term oncologic outcomes following neoadjuvant therapy [18], constituting a further source of residual confounding in the exploratory survival analyses. Finally, the high pathological axillary nodal-positivity rate (92.4%) indicates an enriched, higher-risk, predominantly node-positive cohort reflecting era-specific neoadjuvant referral practice, which limits the external validity of the findings to contemporary, more broadly applied neoadjuvant populations.

## 5. Conclusions

In this NAC-treated retrospective cohort, the numerically higher unadjusted pCR rate observed in HER2-low compared with HER2-zero tumors did not retain statistical significance after multivariable adjustment for hormone receptor expression and tumor grade, and exploratory analyses did not demonstrate significant OS or DFS differences between HER2-low and HER2-zero tumors. However, the wide confidence intervals across all analyses preclude firm conclusions about either the presence or the absence of a true effect. HER2-positive tumors showed higher odds of pCR, although this finding should be interpreted in the context of HER2-directed therapy exposure. The integrated pattern is most cautiously interpreted as a failure to demonstrate a pCR or survival profile for HER2-low disease that is clearly distinct from HER2-zero disease after adjustment in this small cohort, rather than as evidence of biological equivalence. An exploratory hormone receptor-stratified analysis further indicated that the apparent pooled HER2-low pCR advantage was confined to the hormone receptor-negative subgroup, with HER2-low tumors achieving no pathological complete responses among hormone receptor-positive patients, a pattern directionally consistent with the published literature. This conclusion is restricted to the standard NAC setting; in the metastatic landscape, T-DXd has redefined the actionability of HER2-low expression [3]. Future prospective neoadjuvant studies powered for HR-stratified analyses will be required to fully characterize the prognostic relevance and treatment-response implications of HER2-low expression.

## Figures and Tables

**Table 1:** Clinicopathological features according to HER2 status.

Variable	HER2-Low (n = 17)	HER2-Zero (n = 66)	HER2-Positive (n = 35)	Total (n = 118)	*p*
Tumor grade, n (%)					
Grade 1	0 (0.0)	5 (7.7)	0 (0.0)	5 (4.3)	
Grade 2	10 (62.5)	38 (58.5)	15 (42.9)	63 (54.3)	0.084
Grade 3	6 (37.5)	22 (33.8)	20 (57.1)	48 (41.4)	
ER expression, n (%)					
0–10%	6 (35.3)	13 (19.7)	16 (45.7)	35 (29.7)	
10–50%	0 (0.0)	7 (10.6)	7 (20.0)	14 (11.9)	0.006 *
≥50%	11 (64.7)	46 (69.7)	12 (34.3)	69 (58.5)	
PR expression, n (%)					
0–10%	10 (58.8)	35 (53.0)	31 (88.6)	76 (64.4)	
10–50%	3 (17.6)	12 (18.2)	0 (0.0)	15 (12.7)	0.008 *
≥50%	4 (23.5)	19 (28.8)	4 (11.4)	27 (22.9)	
Axillary nodal status, n (%)					
Negative	2 (11.8)	5 (7.6)	2 (5.7)	9 (7.6)	
Positive	15 (88.2)	61 (92.4)	33 (94.3)	109 (92.4)	0.702

ER, estrogen receptor; PR, progesterone receptor. * *p* < 0.05 statistically significant. Grade data available for 116 patients.

**Table 2:** Response to neoadjuvant chemotherapy according to HER2 status.

Variable	HER2-Low (n = 17)	HER2-Zero (n = 66)	HER2-Positive (n = 35)	Total (n = 118)	*p*
Radiological clinical response, n (%)					
Complete response	6 (35.3)	15 (22.7)	14 (40.0)	35 (29.7)	
Partial response	10 (58.8)	40 (60.6)	21 (60.0)	71 (60.2)	0.040
Stable disease	1 (5.9)	11 (16.7)	0 (0.0)	12 (10.2)	
Pathological response, n (%)					
pCR (ypT0/Tis ypN0)	5 (29.4)	8 (12.1)	16 (45.7)	29 (24.6)	
Non-pCR	12 (70.6)	58 (87.9)	19 (54.3)	89 (75.4)	0.001 *

pCR, pathological complete response (ypT0/Tis ypN0). * *p* < 0.05 statistically significant. Comparison by Fisher’s exact test (Monte Carlo).

**Table 3:** Multivariable logistic regression—predictors of pathological complete response.

Variable	OR	95% CI	*p*
HER2-Low vs. HER2-Zero	2.65	0.60–11.75	0.201
HER2-Positive vs. HER2-Zero	4.37	1.46–13.10	0.008 *
Age (years)	1.01	0.97–1.05	0.750
ER expression (ordinal: 0/1/2)	0.65	0.35–1.20	0.167
PR expression (ordinal: 0/1/2)	0.62	0.26–1.49	0.283
Tumor grade	2.63	0.97–7.14	0.059

Reference category: HER2-zero. Covariates: HER2 subgroup, age, ER expression (ordinal 0/1/2), PR expression (ordinal 0/1/2), tumor grade. *n* = 116 (2 excluded: missing grade). * *p* < 0.05. OR, odds ratio; CI, confidence interval.

**Table 4:** Hormone receptor-stratified pathological complete response by HER2 status (descriptive, exploratory).

	HER2-Zero	HER2-Low	HER2-Positive
HR-positive (*n* = 83)	4/53 (7.5%)	0/11 (0.0%)	9/19 (47.4%)
HR-negative (*n* = 35)	4/13 (30.8%)	5/6 (83.3%)	7/16 (43.8%)

Hormone receptor-positive was defined as ER or PR > 10%. Subgroup numbers are small; results are descriptive and hypothesis-generating only.

**Table 5:** Survival outcomes according to HER2 status (Kaplan–Meier, exploratory).

Outcome	HER2-Low (*n* = 17)	HER2-Zero (*n* = 66)	HER2-Positive (*n* = 35)	Overall	Log-Rank *p*
Overall Survival					
Events, *n* (%)	3 (17.6)	10 (15.2)	4 (11.4)	17 (14.4)	0.567
Mean OS (months)	88.0	115.7	94.8	111.5	
95% CI	69.5–106.5	103.9–127.5	80.0–109.6	101.1–121.8	
Median OS (months)	100.2	133.2	NR	133.2	
Disease-Free Survival (recurrence)					
Events, *n* (%)	4 (23.5)	15 (22.7)	7 (20.0)	26 (22.0)	0.901
Mean DFS (months)	49.8	55.9	53.0	54.2	
95% CI	15.5–84.0	40.6–71.1	39.7–66.2	43.7–64.6	
Median DFS (months)	35.0	46.3	46.3	45.5	

NR, not reached. DFS analysis includes patients with recurrence events (*n* = 26). Breslow *p* = 0.815 (OS), *p* = 0.857 (DFS). Survival analyses presented as exploratory secondary outcomes.

**Table 7:** Summary of statistical significance across all univariate analyses.

Variable	Statistical Test	Test Statistic	*p*
Pathological complete response (pCR)	Pearson χ^2^	14.175	0.001 *
Radiological clinical response	Fisher’s exact	—	0.040
ER expression distribution	Pearson χ^2^	14.526	0.006 *
PR expression distribution	Pearson χ^2^	13.926	0.008 *
Tumor grade distribution	Pearson χ^2^	8.224	0.084
Overall survival (OS)	Log-rank	1.133	0.567
Disease-free survival (DFS)	Log-rank	0.208	0.901
Mortality rate	Pearson χ^2^	0.426	0.808

* *p* < 0.05 statistically significant. All comparisons between HER2-low, HER2-zero, and HER2-positive subgroups (*n* = 118).

## Data Availability

The data presented in this study are available on reasonable request from the corresponding author. The data are not publicly available due to institutional and patient privacy restrictions.

## Supplementary Materials

The following supporting information can be downloaded at: https://www.mdpi.com/article/10.3390/medicina62071261/s1, Table S1: Multivariable Cox proportional-hazards models for overall survival (OS) and disease-free survival (DFS), excluding pathological complete response (pCR) as a covariate (sensitivity analysis).

## Abbreviations

The following abbreviations are used in this manuscript: AJCC, American Joint Committee on Cancer; ASCO/CAP, American Society of Clinical Oncology/College of American Pathologists; CI, confidence interval; DFS, disease-free survival; ER, estrogen receptor; FISH, fluorescence in situ hybridization; HER2, human epidermal growth factor receptor 2; HR, hazard ratio (in survival analysis); HR, hormone receptor (in tumor classification); IHC, immunohistochemistry; NAC, neoadjuvant chemotherapy; OR, odds ratio; OS, overall survival; pCR, pathological complete response; PR, progesterone receptor; T-DM1, trastuzumab emtansine; T-DXd, trastuzumab deruxtecan; TNBC, triple-negative breast cancer.
