# Supplementary material for: HER2-Low Versus HER2-Zero Breast Cancer in the Neoadjuvant Setting: Pathological Complete Response and Exploratory Survival Outcomes in a Single-Center Cohort
Source: Medicina (Kaunas). 2026 Jun 30;62(7):1261. doi: 10.3390/medicina62071261 (PMC13413514; doi:10.3390/medicina62071261)
Supplement: Supplementary file 1 [file medicina-62-01261-s001.zip › medicina-4360936-supplementary.pdf]

Supplementary Table S1. Multivariable Cox proportional-hazards models for overall survival (OS) and disease-free survival (DFS), excluding pathological complete response (pCR) as a covariate (sensitivity analysis).

	Overall Survival (OS)			Disease-Free Survival (DFS)		
**Covariate**	**HR**	**95% CI**	**p**	**HR**	**95% CI**	**p**
**HER2-low (vs HER2-zero)**	2.56	0.63–10.42	0.191	1.35	0.43–4.24	0.612
**HER2-positive (vs HER2-zero)**	0.86	0.21–3.57	0.836	1.10	0.42–2.87	0.851
**Age (years)**	0.97	0.92–1.02	0.250	0.98	0.95–1.01	0.250
**ER expression (ordinal)**	0.91	0.42–1.96	0.807	0.84	0.49–1.44	0.529
**PR expression (ordinal)**	2.19	1.09–4.40	0.027	1.57	0.92–2.68	0.100
**Tumor grade**	3.22	0.99–10.50	0.053	1.71	0.78–3.73	0.182

OS model: n = 116, 17 events. DFS model: n = 115, 29 events. Reference category for HER2 subgroup: HER2-zero. ER and PR expression entered as ordinal variables (0/1/2). pCR was not included as a covariate. HR, hazard ratio; CI, confidence interval; pCR, pathological complete response.

Interpretation. This sensitivity model corresponds to the primary multivariable Cox models in Table 5 with the pCR term removed. Excluding pCR did not change the substantive findings: no HER2 subgroup was significantly associated with OS or DFS, and both HER2-low and HER2-positive remained non-significant. This confirms that the absence of a HER2 subgroup survival effect is not an artefact of adjustment for pCR. As in Table 5, the very small number of events relative to covariates yields wide confidence intervals, and the hazard-ratio point estimates should be interpreted as exploratory only.
